# Supplementary material for: PEGylation of Terminal Ligands as a Route to Decrease the Toxicity of Radiocontrast Re6-Clusters
Source: Int J Mol Sci. 2023 Nov 21;24(23):16569. doi: 10.3390/ijms242316569 (PMC10706756; doi:10.3390/ijms242316569)
Supplement: Supplementary file 1 [file ijms-24-16569-s001.zip › ijms-2717545-supplementary.pdf]

Supporting Information

PEGylation of Terminal Ligands as a Route to Decrease the Toxicity of Radiocontrast Re_6-Clusters

Aleksei S. Pronin [1], Tatiana N. Pozmogova [1], Yuri A. Vorotnikov [1,*], Georgy D. Vavilov [2], Anton A. Ivanov [1], Vadim V. Yanshole [3,4], Alphiya R. Tsygankova [1,5], Tatiana Ya. Gusel'nikova [1,4], Yuri V. Mironov [1,*] and Michael A. Shestopalov [1]

[1] Nikolaev Institute of Inorganic Chemistry SB RAS, 3 Acad. Lavrentiev Ave., Novosibirsk 630090, Russia; pronin@niic.nsc.ru (A.S.P.); tnp_post@mail.ru (T.N.P.); ivanov338@niic.nsc.ru (A.A.I.); alphiya@niic.nsc.ru (A.R.T.); tguselnikova@niic.nsc.ru (T.Y.G.); shtopy@niic.nsc.ru (M.A.S.)
[2] National Medical Research Center for Circulation Pathology n.a. Academician E.N. Meshalkin, 15 Rechkunovskaya St., Novosibirsk 630055, Russia; frost20@yandex.ru
[3] International Tomography Center SB RAS, 3a Institutskaya St., Novosibirsk 630090, Russia; vadim.yanshole@tomo.nsc.ru
[4] Department of Physics, Novosibirsk State University, 2 Pirogova Str., Novosibirsk 630090, Russia
[5] Department of Natural Sciences, Novosibirsk State University, 2 Pirogova Str., Novosibirsk 630090, Russia
* Correspondence: vorotnikov@niic.nsc.ru (Y.A.V.), yuri@niic.nsc.ru (Y.V.M.)

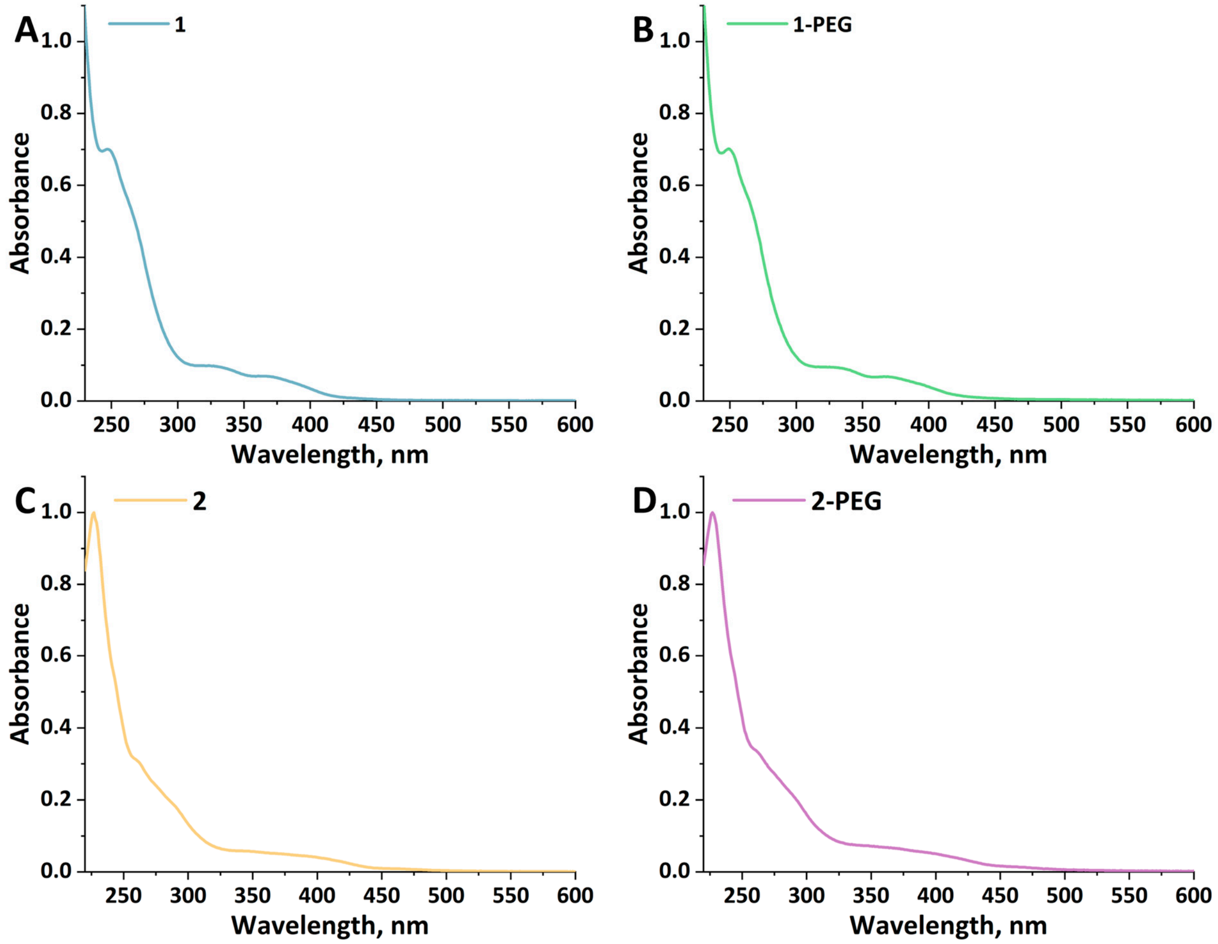

Figure S1. UV-vis spectra of 1 (A), 1-PEG (B), 2 (C), and 2-PEG (D) in water.

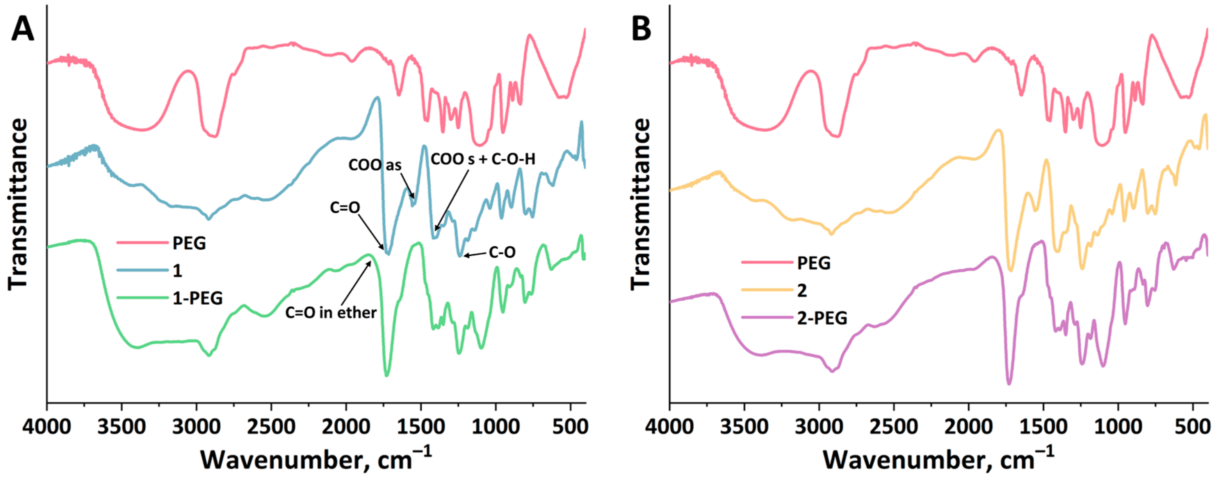

Figure S2. FTIR spectra of 1 and 1-PEG (A) and 2 and 2-PEG (B) in comparison with PEG-400.

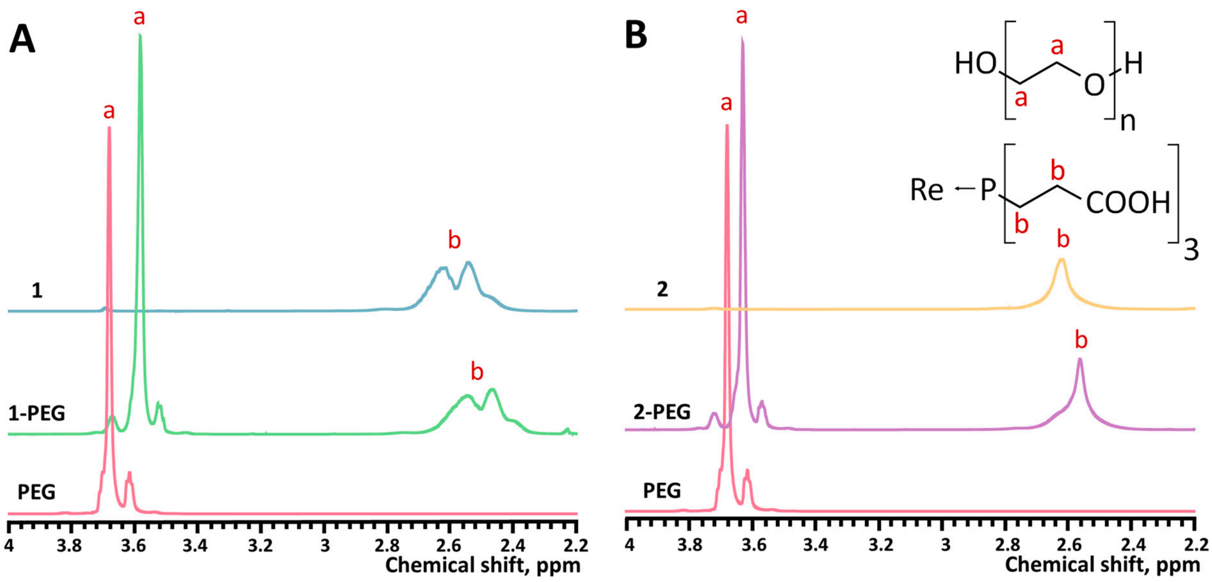

Figure S3. ^{1}H-NMR spectra of 1 and 1-PEG (A) and 2 and 2-PEG (B) in D_2O in comparison with PEG-400.

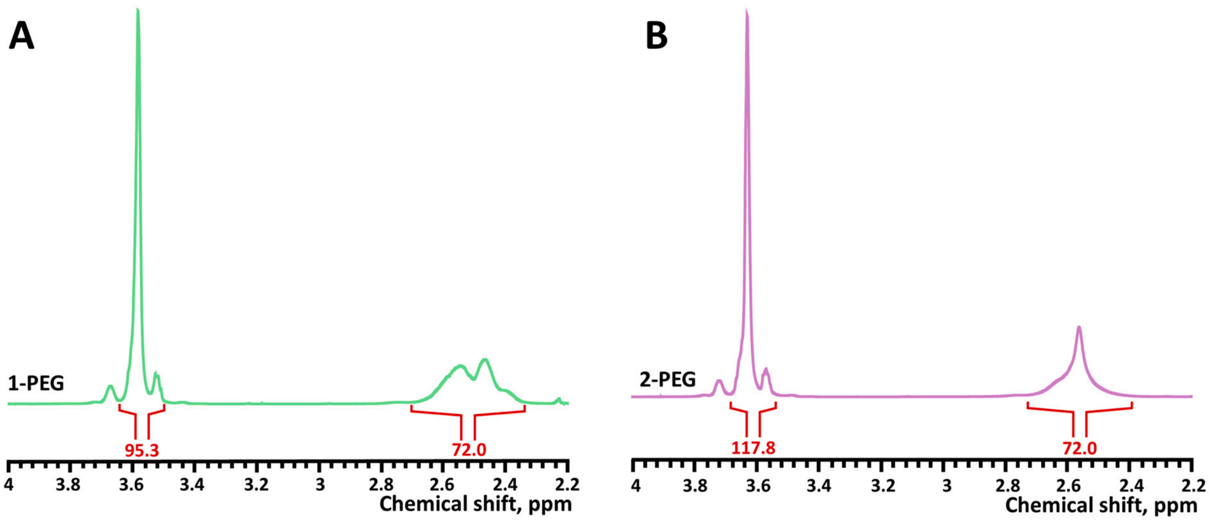

Figure S4. Fragment of NMR spectra of 1-PEG (A) and 2-PEG (B) with integral intensity of PEG and cluster signals.

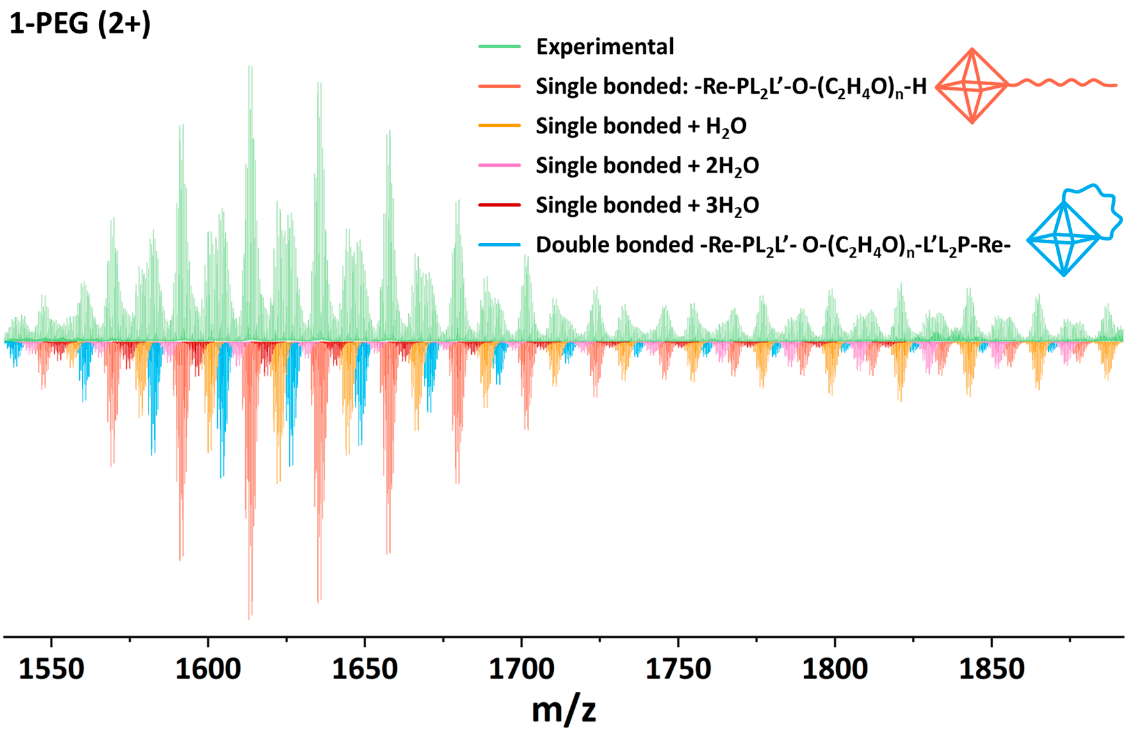

Figure S5. HR-ESI-MS spectra of the aqueous solutions of 1-PEG (green) in positive mode (2+) and a simulation of PEGylated cluster forms (colored).

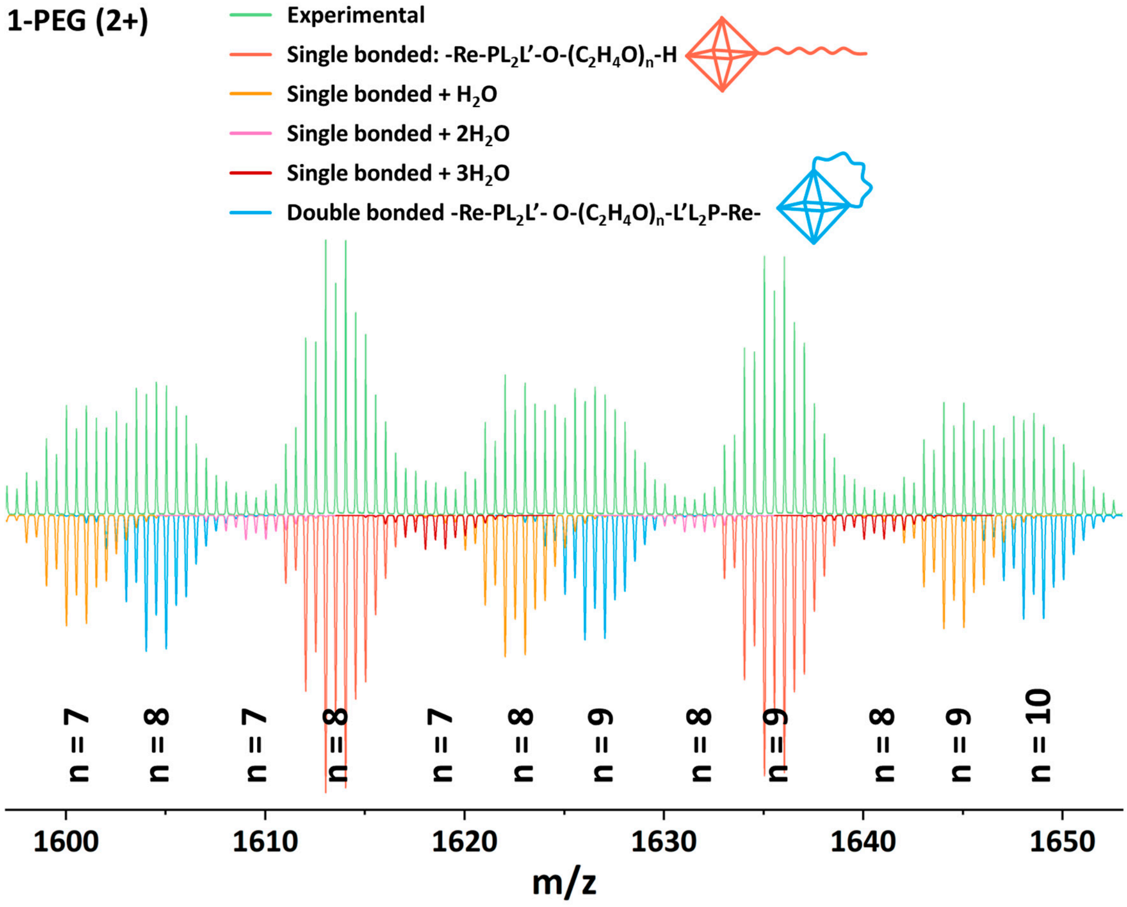

Figure S6. Enlarged fragment of HR-ESI-MS spectra of the aqueous solutions of 1-PEG (green) in positive mode (2+) and a simulation of PEGylated cluster forms (colored).

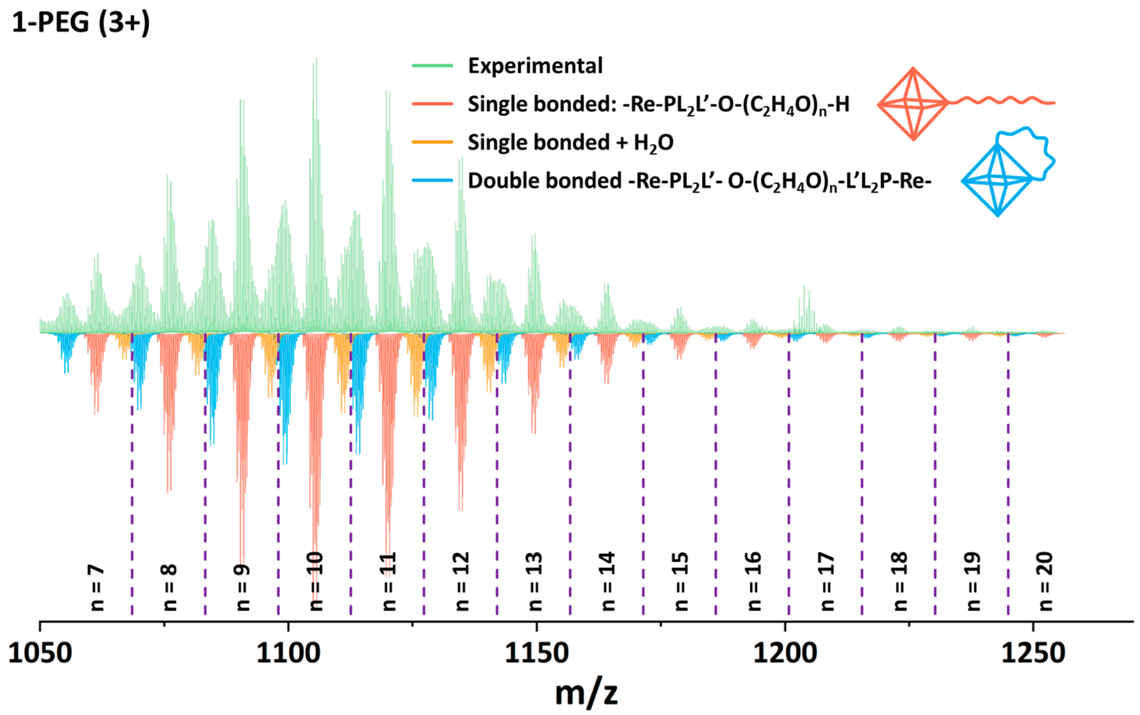

Figure S7. HR-ESI-MS spectra of the aqueous solutions of 1-PEG (green) in positive mode (3+) and a simulation of PEGylated cluster forms (colored).

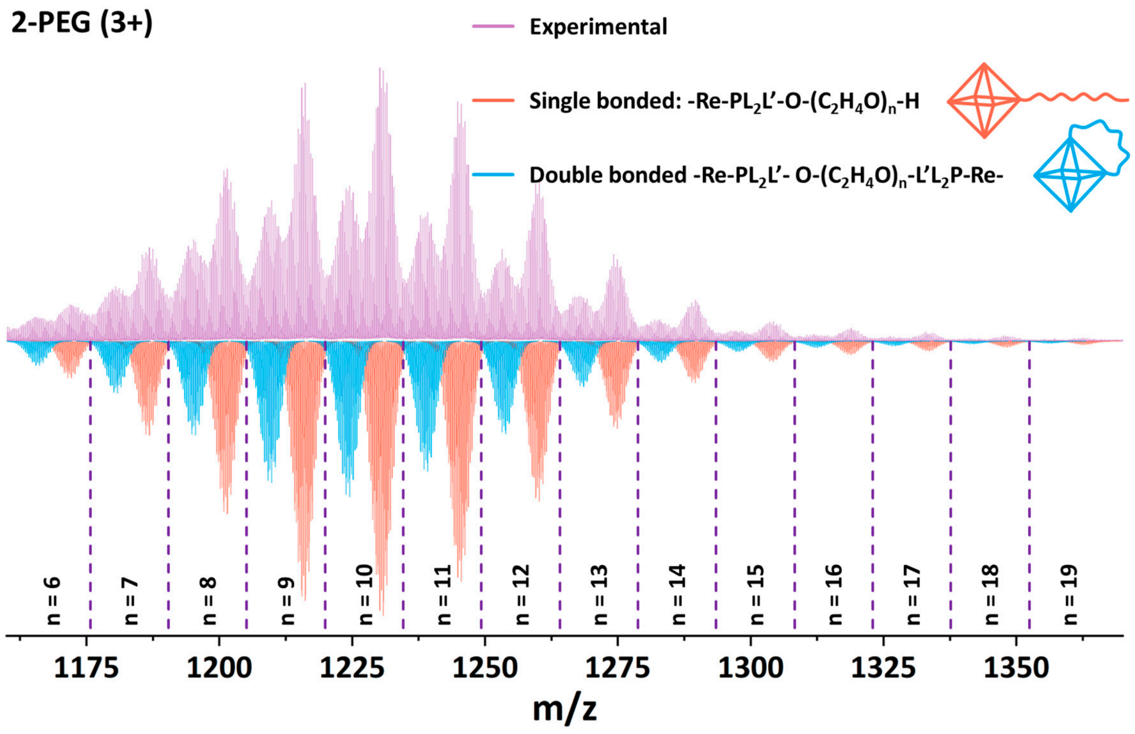

Figure S8. HR-ESI-MS spectra of the aqueous solutions of 2-PEG (pink) in positive mode (3+) and a simulation of PEGylated cluster forms (colored).

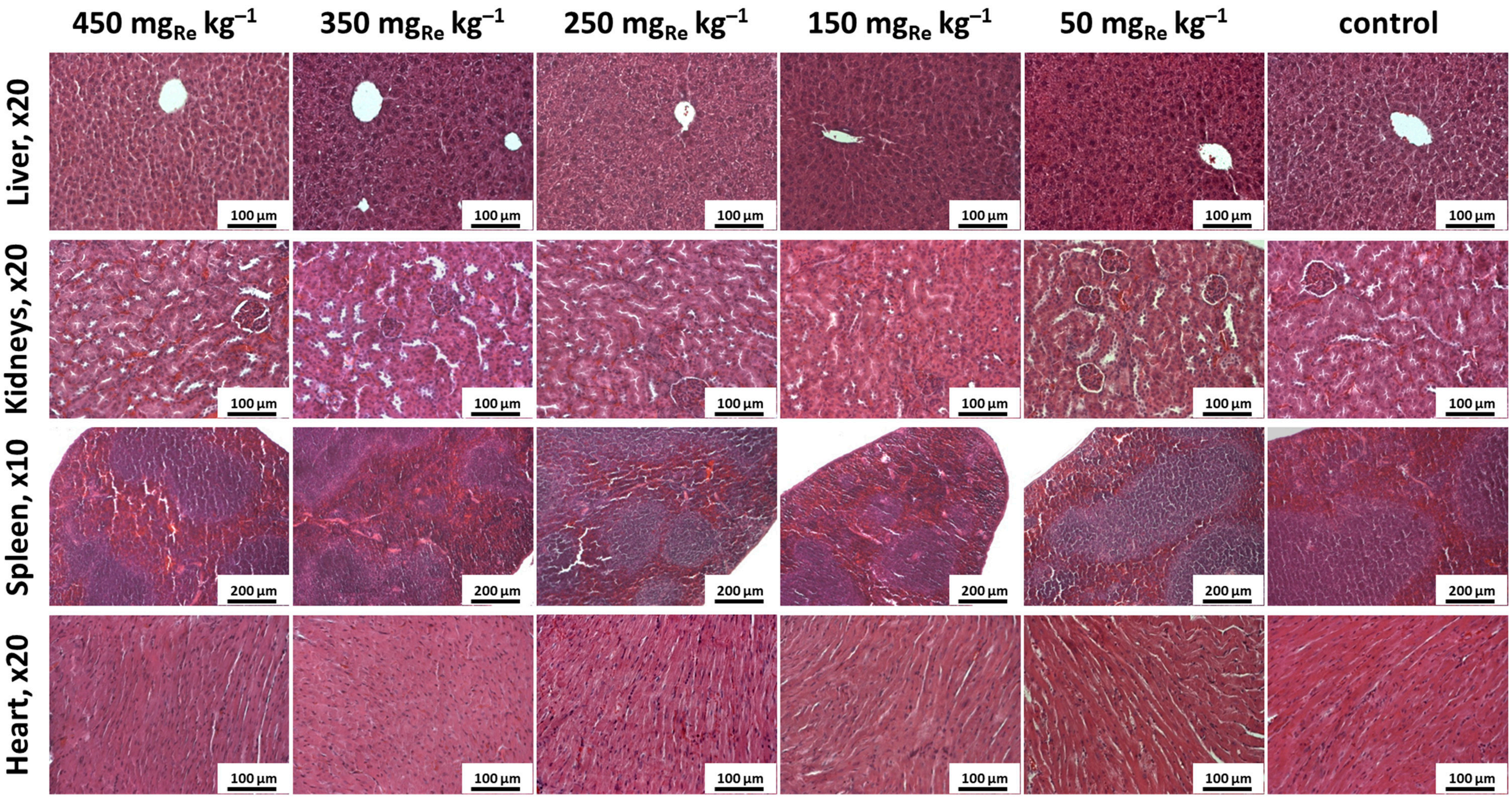

Figure S9. Morphology of mouse organs 2 weeks after intravenous administration of 1-PEG, stained with hematoxylin-eosin.

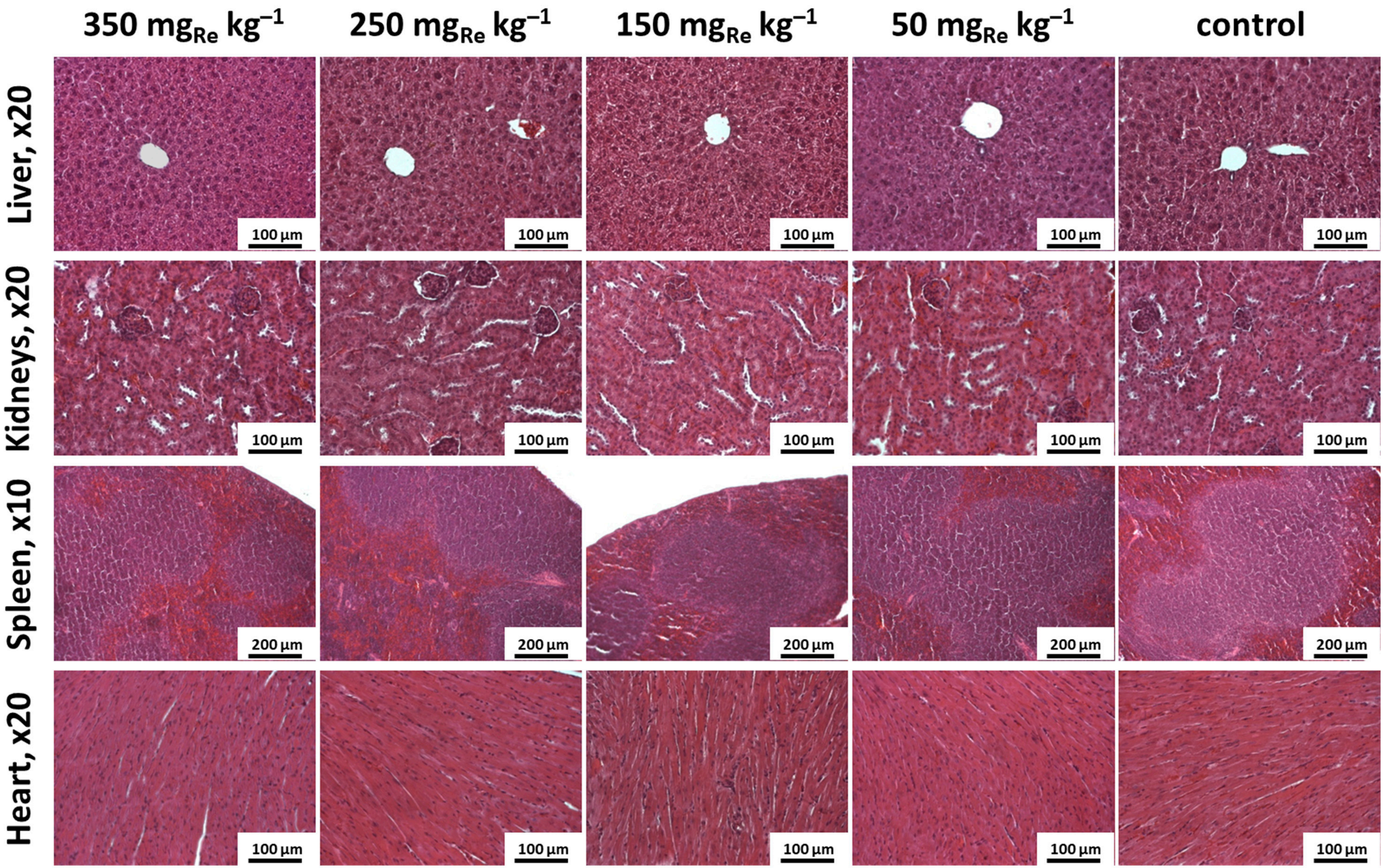

Figure S10. Morphology of mouse organs 2 weeks after intravenous administration of 2-PEG, stained with hematoxylin-eosin.
